# Maternite Hospitals

**Published:** 1881-05

**Authors:** 


Maternite Hospital.—"Is the Maternite, or Lying-in Hospital, on Lombard, between Sharp and Hanover streets, a State or private establishment? If owned by the State, what was its cost? The reasons for asking these questions may be made known hereafter; should your answers not disclose what is wanted———?"

Fraternally yours, J. A.

A. We regret that we are not sufficiently posted to supply the information you seek, as we are not a "professor" in the concern. But an appropriation of $20,000 was made by the legislature for founding a lying-in hospital in Baltimore, about the time of the inception of the establishment alluded to, and other appropriations may have been made to it subsequently, for aught we know to the contrary. The "dean," however, could doubtless supply the required information, as *he* knows what the *net* amount was that went into the establishment from the first legislative appropriation, and probably also any other sums that may have been added since that time. Your further enquiry is legitimate and fair, and we will extend you any assistance we can at the proper time.

